# Embolic Stroke Following an Amniotic Fluid Embolism in the Setting of Patent Foramen Ovale

**DOI:** 10.1155/crog/7954851

**Published:** 2026-06-23

**Authors:** Osamah Khan, Michael DeRosa, Lydia Holden, Jason Hoppe, Alton Hallum, Michael Krew

**Affiliations:** ^1^ Aultman Hospital Department of OB/GYN, Northeast Ohio Medical School, Rootstown, Ohio, USA

## Abstract

Amniotic fluid embolism (AFE) is a rare but devastating complication during pregnancy. We present an instance of an embolic stroke following an AFE in labor with a concurrent patent foramen ovale (PFO). The patient was a 34‐year‐old G4P2 who had an acute seizure event intrapartum. She was taken for an emergent cesarean delivery during which she was noted to have cardiopulmonary compromise. A cesarean hysterectomy was performed due to ongoing DIC and hemorrhage. Postoperatively, she was found to have evidence of thrombotic emboli as well as a PFO. She had a good neurologic recovery with supportive measures even prior to discharge from the hospital and has continued to do well. When more common etiologies of intrapartum seizures have been excluded, providers should maintain a high index of suspicion for embolic stroke.

## 1. Introduction

Amniotic fluid embolism (AFE) is a rare and potentially fatal complication of pregnancy that can occur during labor, delivery, or immediately postpartum. AFE is estimated to occur in 1.9–6.1 per 100,000 deliveries (1, 2). Most cases occur during labor. The pathogenesis is thought to involve an anaphylactoid‐type reaction to the presence of amniotic fluid in maternal circulation. The initial presentation is usually sudden onset of hypoxia and hemodynamic instability secondary to cardiovascular collapse due to right ventricular outflow tract obstruction (3). The amniotic fluid also leads to dysregulated activation of the clotting cascade, leading to disseminated intravascular coagulopathy (DIC). Early recognition is critical, as unrecognized AFE can quickly become fatal. Management is supportive and involves prompt resuscitative efforts, expedited delivery, and correction of underlying coagulopathy. Of important interest in this case is the presence of a patent foramen ovale (PFO). A PFO is a congenital defect between the right and left atria that can persist into adulthood. A right to left shunt across the PFO may develop in situations where the pressure gradient is increased, such as an AFE. This can lead to a paradoxical embolus causing an embolic stroke in the brain (4). We report a unique presentation of AFE in which the patient had an embolic stroke at the time of her AFE due to a previously undiagnosed PFO.

## 2. Case Presentation

The patient was a 34‐year‐old G4P2 who was admitted for induction of labor due to chronic hypertension (cHTN) at 38/0 weeks gestational age. Pregnancy was complicated by cHTN, Class III obesity, migraines, large‐for‐gestational‐age (LGA) infant, history of intrahepatic cholestasis of pregnancy, and history of T‐cell lymphoma. The patient′s cHTN was well controlled without medications. She had no previously documented abdominal/gynecological surgery and had had two uncomplicated previous vaginal deliveries. Her prenatal course was unremarkable.

Induction was initiated with oxytocin. Fetal heart tracing was Category 1 at that time. Initial vital signs were within normal limits. The patient made cervical change to 4‐cm dilation prior to spontaneous rupture of membranes (SROM). SROM occurred while the patient was in the bathroom; she was then helped back to bed by her nurse, who witnessed seizure‐like activity after loss of consciousness. An emergency call light was activated, and the attending physician reported to bedside. Her unusual seizure‐like activity after loss of consciousness was initially presumed to be due to eclampsia, and a 6‐g magnesium bolus was ordered. Subsequently, a fetal scalp electrode was placed, due to difficulty tracing fetal heart tones that demonstrated prolonged fetal bradycardia. At this time, the decision was made to proceed to the OR in a STAT fashion for emergency cesarean delivery. She underwent general anesthesia for protection of her airway and did not have an epidural in place. A delay in magnesium therapy occurred due to the loss of patient IV access, but it was quickly re‐established, and magnesium was started intraoperatively. Oozing was noted around the IV sites even before the start of surgery. After delivery and closure of hysterotomy, the uterus was noted to be atonic contributing to resultant postpartum hemorrhage. Multiple uterotonics were given intraoperatively. Despite being intubated, the patient was noted to by hypoxemic down to the 60s and required initiation of pressors due to hypotension. Delivery was complicated by a left uterine extension down to the level of the vagina. O′Leary sutures were placed on the left to attempt to control continued brisk bleeding but ultimately failed. Significant lab derangements were noted consistent with DIC (fibrinogen < 35 and aPTT > 200) with new onset hypoxemia and hypotension, thus raising suspicion for AFE. Massive transfusion protocol was initiated. Gynecologic Oncology was consulted for additional surgical support. Cesarean hysterectomy was performed as a final effort to control bleeding and for maternal resuscitative benefit; estimated blood loss was 3000 mL. Improvement of her hypoxemia and hypotension was noted following cesarean hysterectomy.

**Figure 1:**
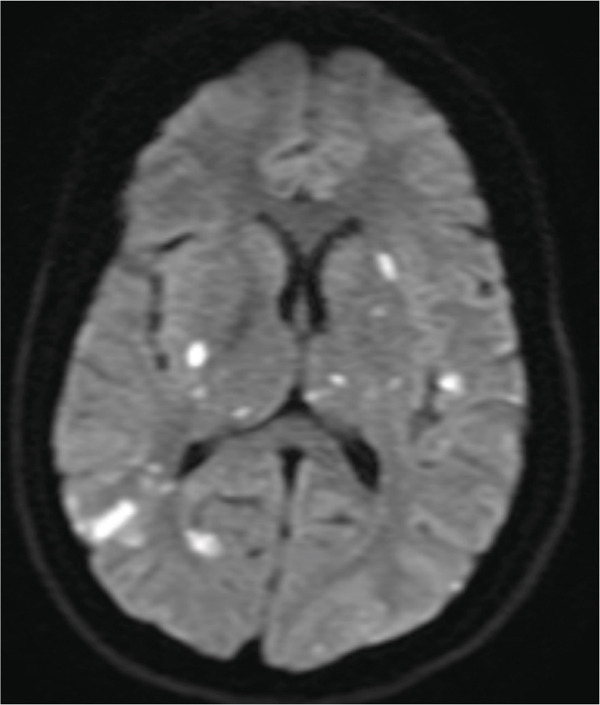
MRI axial cross section without contrast demonstrating diffuse infarcts.

The patient was moved to the SICU after surgery for continued stabilization. Continued significant lab derangements were noted, including a fibrinogen level of 230, a troponin level of 3900, and lactic acidosis with a lactate level of 3.1. She was afebrile from the time of her induction and throughout her postoperative course. She was taken back to the OR the next morning by Gynecologic Oncology due to increased JP drain output and had an intra‐abdominal hematoma washout on postoperative day (POD) 1. She received 13 units of pRBCs, 7 units of FFP, 3 units of platelets, and 4 units of cryoprecipitate by POD 1. On POD 2, the patient was extubated. She was noted to be nonverbal and minimally responsive to painful stimuli. Cranial imaging was ordered due to her altered mental status. On POD 3, the patient underwent a CT Brain which showed acute infarcts in the right occipital lobe. An MRI revealed acute supra and infratentorial infarcts. Specifically, there was involvement of the left cerebellum/left cerebellar vermis as well as the bilateral frontal, parietal, temporal, and occipital lobes. Infarcts were also noted in the bilateral extreme capsules, right insular cortex, bilateral thalami, bilateral basal ganglia, bilateral precentral, and postcentral gyri (Figure 1). Transthoracic echocardiogram was obtained and initially showed normal EF with no shunts. A follow‐up transthoracic echocardiogram with bubble study also failed to identify a shunt in the atrial septum. Follow‐up transesophageal echocardiogram demonstrated an increase in right atrial pressure induced by provocative maneuvers indicating a right‐to‐left atrial level shunt. This diagnosis of PFO was suspected to be the contributing factor to her embolic infarcts. Neurology recommended an MRA/MRV study of the head and a hypercoagulability workup, which were negative. On POD 4, lower extremity ultrasound was performed which showed no evidence of DVT bilaterally. The patient went on to make significant neurological recovery. She was started on Lovenox for a segmental/subsegmental pulmonary embolism. She had marked improvement in her mentation prior to discharge. She was discharged from the hospital on POD 9. She was admitted two subsequent times for pelvic hematoma. She required an additional transfusion of 2 units pRBCs, and her anticoagulation was temporarily held; the hematoma was managed expectantly and did not require surgical intervention. She is recovering well and follows with cardiology outpatient for possible closure of PFO. Follow‐up histopathologic examination of the patient′s placenta and uterus did not reveal any significant findings.

## 3. Discussion

Despite being an exceedingly rare event, AFEs represent a significant portion of maternal mortality, as much as 10% according to one study (5). There is only one other reported incidence of stroke following an AFE that we were able to locate following a thorough literature search of PubMed and Google Scholar using key phrases such as “stroke” and “amniotic fluid embolism.” Woo et al. report a similar case of ischemic stroke following an AFE in the setting of a PFO (6). Kumar et al. report a case of paradoxical emboli following an AFE; however, the embolus did not travel to the brain and cause a stroke (7).

AFE remains a poorly understood phenomenon, and more research is needed to fully elucidate the pathophysiology and potential risk factors. The release of vasoconstrictive/inflammatory factors is hypothesized to lead to bronchoconstriction which increases strain on the right ventricle (3). In the case of this patient, the increased pressure on the interatrial septum likely led to right‐to‐left shunting across a preexisting PFO. This allowed an embolus from the venous circulation to cross over to arterial circulation and eventually led to the patient′s stroke. Initial diagnosis can be challenging, as the differential is broad: eclampsia, maternal cardiac arrest, epilepsy, and other life‐threatening conditions must be ruled out. Prompt recognition of DIC led to early initiation of massive transfusion and cesarean hysterectomy.

Our case is atypical in that the first sign of an AFE event was fulminant seizure‐like activity, which is more commonly associated with eclampsia or epilepsy. The patient was initially noted to be nonverbal and minimally responsive to commands following extubation; however, it was initially unclear if this was due to the effects of anesthesia or from a neurological insult. Only subsequent imaging confirmed the presence of cerebral watershed infarcts. At the time of this report, the patient has continued to recover well from her AFE, with minimal residual neurologic deficits noted during her recent appointments. It is unknown what level of neurologic recovery is to be expected in such cases; in the only previously reported case of stroke following AFE, the patient′s neurologic recovery was not addressed. In general, approximately 10%–30% of patients who experience a stroke will develop disability of at least moderate level within 90 days (8).

We recommend that all providers be vigilant for cerebrovascular compromise following AFE or other intrapartum event and initiate appropriate workup especially in the setting of mental status changes.

## Author Contributions

Osamah Khan: conceptualization, writing – original draft. Michael DeRosa: writing – review and editing. Lydia Holden: writing – review and editing. Jason Hoppe: supervision. Alton Hallum III: supervision. Michael Krew: supervision.

## Funding

No funding was received for this manuscript.

## Ethics Statement

The Aultman Hospital IRB approved this case report.

## Consent

Appropriate informed consent was obtained from the patient for publication of this case report.

## Conflicts of Interest

The authors declare no conflicts of interest.

## Data Availability

The data that support the findings of this case report are not publicly available due to patient privacy and ethical restrictions.
